# Artificial intelligence in pharmacy practice in the Middle East: a scoping review of applications, adoption, barriers, and future directions

**DOI:** 10.3389/fdgth.2026.1951084

**Published:** 2026-10-09

**Authors:** Faten Alhomoud

**Affiliations:** Department of Pharmacy Practice, College of Pharmacy, Imam Abdulrahman Bin Faisal University, Dammam, Saudi Arabia

**Keywords:** artificial intelligence, chatGPT, digital health, large language models, Middle East, pharmacists, pharmacy practice, scoping review

## Abstract

**Background:**

Artificial intelligence (AI) is transforming pharmacy practice by supporting medication management, clinical decision-making, patient education, and pharmacy operations. Although AI research in the Middle East has expanded rapidly, the evidence remains fragmented across countries, technologies, and practice settings. This scoping review aimed to systematically map the current evidence on AI applications, adoption, perceived benefits, implementation barriers, and ethical and regulatory considerations in pharmacy practice across the Middle East.

**Methods:**

A scoping review was conducted according to the Joanna Briggs Institute methodology and reported following the PRISMA-ScR guidelines. PubMed, Scopus, and Web of Science were systematically searched for English-language studies investigating AI in pharmacy practice in Middle Eastern countries. Two reviewers independently screened studies and extracted data. Findings were synthesized narratively according to AI applications, utilization, pharmacists' awareness and attitudes, perceived benefits, implementation barriers, ethical and regulatory issues, and future research priorities.

**Results:**

A total of 15 studies published between 2023 and 2026, involving 8,117 participants across multiple Middle Eastern countries, were included. Most studies were cross-sectional surveys (93.3%) evaluating pharmacists' awareness, perceptions, and readiness to adopt AI. ChatGPT and large language models were the most frequently investigated technologies, primarily supporting medication information, patient counselling, medication management, and clinical decision support, while AI-enabled robotics focused on dispensing and pharmacy operations. Pharmacists generally demonstrated high awareness and positive attitudes toward AI, and some studies reported previous or individual use; however, evidence of routine use and organization-level implementation remained limited. Perceived benefits included improved clinical decision-making, medication safety, and workflow efficiency, whereas major barriers included inadequate AI training, limited technological infrastructure, implementation costs, organizational readiness, and concerns regarding privacy, cybersecurity, algorithmic bias, transparency, and professional accountability.

**Conclusion:**

AI has considerable potential to enhance pharmacy practice across the Middle East, but implementation remains at an early stage, with the available literature predominantly reflecting pharmacists' perceptions of anticipated benefits rather than evidence of demonstrated clinical effectiveness. Strengthening AI education, digital infrastructure, governance frameworks, and implementation research will be essential to support the safe, ethical, and evidence-based integration of AI into pharmacy practice.

## 1 Introduction

Artificial intelligence (AI) is rapidly transforming healthcare by enabling computer systems to perform tasks that traditionally require human intelligence, including learning, reasoning, pattern recognition, language processing, and clinical decision-making. Recent advances in machine learning, deep learning, natural language processing, large language models (LLMs), and generative AI have accelerated the integration of AI into healthcare delivery, creating new opportunities to improve efficiency, support clinical decisions, enhance patient safety, and optimize healthcare resource utilization. Among these technologies, generative AI tools such as ChatGPT have attracted considerable attention for their ability to retrieve, synthesize, and generate human-like responses to complex clinical and pharmaceutical questions, potentially reshaping how healthcare professionals access and use medical information (1–4).

Pharmacy practice is among the healthcare disciplines most likely to benefit from AI implementation, as pharmacists routinely manage large volumes of medication-related information and make complex clinical decisions (3–5). AI technologies have demonstrated potential applications across multiple areas of pharmacy practice, including medication information services, clinical decision support, medication dispensing, prescription verification, drug interaction screening, medication therapy management, pharmacovigilance, inventory management, workflow optimization, and personalized pharmaceutical care (3–6). More recently, LLMs and generative AI have expanded these capabilities by supporting medication counseling, clinical documentation, evidence retrieval, patient education, and drug information provision. These technologies have been proposed as having the potential to reduce medication errors, improve operational efficiency, support clinical decision-making, and allow pharmacists to devote more time to direct patient care (3–6).

Despite these promising developments, successful AI implementation depends not only on technological capability but also on healthcare professionals' readiness to adopt and use these technologies appropriately (7–9). Pharmacists remain central to medication management and pharmaceutical care, and their acceptance of AI is critical for its successful integration into routine practice (8–10). Previous studies have suggested that while pharmacists generally recognize the potential benefits of AI (8–11), concerns remain regarding the accuracy of AI-generated recommendations (7, 10, 12), patient safety (13–15), data privacy, cybersecurity, legal liability, ethical considerations (10, 14, 16), transparency of AI algorithms (14, 16), insufficient training (9, 17–19), and the possibility of replacing aspects of pharmacists' professional roles (10, 16, 20, 21). Understanding these perceptions is essential for developing implementation strategies that ensure AI complements rather than replaces professional pharmaceutical judgment.

The Middle East has experienced rapid digital transformation in healthcare over the past decade, supported by national digital health initiatives and governmental strategies such as Saudi Vision 2030, the UAE Digital Health Strategy, and comparable digital transformation programs across the region (22–24). These initiatives have accelerated the adoption of electronic health records, telehealth, clinical decision support systems, robotics, and AI-enabled healthcare services (22–24). Consequently, an increasing number of studies have investigated AI within pharmacy practice across Middle Eastern countries, including Saudi Arabia (11, 17, 20), Jordan (7, 8), the United Arab Emirates (10, 12, 15), Egypt (13), Qatar (18), Syria (19), and multinational studies involving the Middle East (9, 16). Recent research has explored pharmacists' awareness, knowledge, attitudes (8, 9, 11), willingness to adopt AI, actual AI utilization (9, 11, 17), perceptions of ChatGPT (7, 10, 13) and other LLMs, ethical concerns (16), implementation barriers (8, 18, 19), robotic technologies (15), and AI applications across both community and hospital pharmacy settings. Collectively, these studies provide valuable insights into the evolving landscape of AI adoption in pharmacy practice within the region (9, 10, 16, 24).

Despite the growing number of studies, the available evidence remains scattered across different AI technologies, pharmacy settings, countries, and research objectives (7–10, 15, 16). Existing studies have predominantly focused on individual countries (7, 10, 11, 13, 18, 19) or specific AI applications (7, 12, 15, 17), while others have examined pharmacists' perceptions (8–11), ethical considerations (16), or implementation challenges (8, 18, 19) independently. To our knowledge, based on searches of PubMed, Scopus, and Web of Science and a literature check of relevant reviews, no previous scoping review has specifically synthesized evidence on AI in pharmacy practice across the Middle East. Such a synthesis is necessary to provide an overview of current AI applications, identify patterns of adoption, summarize perceived benefits and implementation barriers, evaluate ethical considerations, and highlight evidence gaps requiring future investigation.

A scoping review is particularly appropriate because AI in pharmacy practice is an emerging, rapidly evolving field characterized by diverse technologies, heterogeneous study designs, and varied practice settings. Unlike systematic reviews that primarily evaluate intervention effectiveness, scoping reviews are designed to comprehensively map the available evidence, identify key concepts, summarize the range of existing research, and inform future research priorities, policy development, and implementation strategies (25–27).

Therefore, this scoping review aims to systematically map and synthesize the current evidence on artificial intelligence in pharmacy practice across the Middle East region by identifying current AI applications, assessing pharmacists' awareness, knowledge, attitudes, willingness, and utilization of AI technologies, summarizing reported benefits and implementation challenges, exploring ethical and regulatory considerations, and identifying evidence gaps and future directions for research, policy, and pharmacy practice.

Review question: What is the current evidence on the applications, adoption, perceptions, barriers, and future directions of artificial intelligence in pharmacy practice across the Middle East?

## 2 Methods

### 2.1 Design

This scoping review was conducted in accordance with the Joanna Briggs Institute (JBI) methodology for scoping reviews and is reported in accordance with the Preferred Reporting Items for Systematic Reviews and Meta-Analyses extension for Scoping Reviews (PRISMA-ScR) guidelines (26, 27). The JBI framework was used to guide all stages of the review, including the development of the review question, eligibility criteria, literature search, study selection, data extraction, and synthesis of the evidence. The PRISMA-ScR checklist was used to ensure transparent and comprehensive reporting of the review methodology and findings. The review was undertaken to systematically map the existing evidence on the application of artificial intelligence in pharmacy practice across Middle Eastern countries.

### 2.2 Search strategy

A comprehensive literature search was conducted to identify published studies examining the use of artificial intelligence (AI) in pharmacy practice across Middle Eastern countries. For the purposes of this review, the Middle East was operationally defined as comprising Bahrain, Egypt, Iran, Iraq, Israel, Jordan, Kuwait, Lebanon, Oman, Palestine, Qatar, Saudi Arabia, Syria, Turkiye, the United Arab Emirates, and Yemen, consistent with the regional classification used by the Pew Research Center (28). The search strategy was developed according to the Joanna Briggs Institute (JBI) methodology for scoping reviews and was structured using the Population–Concept–Context (PCC) framework.

Three electronic databases were searched: PubMed, Scopus, and Web of Science Core Collection. These databases were selected to provide complementary coverage of the biomedical, pharmacy, and broader multidisciplinary literature relevant to AI in pharmacy practice. A combination of Medical Subject Headings (MeSH), free-text terms, and keywords related to the three main concepts—artificial intelligence, pharmacy practice, and Middle Eastern countries—was used to develop the search strategy. These terms were combined using Boolean operators (AND and OR) to retrieve relevant studies.

The core search concepts and corresponding search terms are presented in Table 1. These terms were adapted as required to the syntax and indexing conventions of PubMed, Scopus, and Web of Science Core Collection. Each database was searched on 30 June 2026, with coverage from database inception to the search date. No restrictions were placed on publication year to ensure comprehensive retrieval of the available literature. Searches were limited to articles published in English. In addition to database searching, the reference lists of all included studies were manually screened to identify any additional eligible articles, consistent with established scoping review methodology.

**Table 1:** Search concepts and terms used to identify studies on artificial intelligence in pharmacy practice across the Middle East.

Search terms for “Artificial intelligence”	And	Search terms for “Pharmacy practice”	And	Search terms for “Middle East”
“Artificial Intelligence” OR “AI” OR “Machine Learning” OR “Deep Learning” OR “Natural Language Processing” OR “NLP” OR “ChatGPT” OR “GPT” OR “Large Language Model” OR “LLM” OR “Generative Artificial Intelligence” OR “Generative AI”		“Pharmacist” OR “Pharmacy” OR “Pharmacy Practice” OR “Clinical Pharmacist” OR “Community Pharmacist” OR “Hospital Pharmacist” OR “Ambulatory Care Pharmacist” OR “Pharmaceutical Care” OR “Pharmaceutical Services” OR “Medication Management”		“Middle East” OR “Saudi Arabia” OR “United Arab Emirates” OR “Bahrain” OR “Kuwait” OR “Oman” OR “Qatar” OR “Jordan” OR “Lebanon” OR “Iraq” OR “Iran” OR “Syria” OR “Palestine” OR “Yemen” OR “Egypt” OR “Turkiye” OR “Israel”

### 2.3 Eligibility criteria

The eligibility criteria were developed using the Participants, Concept, and Context (PCC) framework recommended by the Joanna Briggs Institute (JBI) for scoping reviews, which also informed the development of the search strategy (26). For the purposes of eligibility, a technology was considered within the AI concept when the primary study explicitly described or evaluated it as AI, AI-enabled, or AI-assisted. Robotics and clinical decision-support systems were therefore included only when an AI component was explicitly identified. Routine pharmacy automation, such as conventional automated dispensing or workflow systems without an identified AI component, and conventional rule-based clinical decision-support systems were not considered AI for inclusion. The detailed inclusion and exclusion criteria are presented in Table 2.

**Table 2:** Inclusion and exclusion criteria.

Domain	Inclusion	Exclusion
Participants (P)	Pharmacists or pharmacy services in any pharmacy practice setting, including community, hospital, clinical, ambulatory care, and other pharmacy practice settings.	Studies involving healthcare professionals that do not report pharmacist-specific findings. Studies focusing exclusively on pharmacy students, pharmacy faculty, or pharmacy education
Concept (C)	Original empirical studies investigating the application, implementation, adoption, utilization, awareness, knowledge, attitudes, perceptions, barriers, facilitators, ethical or regulatory considerations, or outcomes of artificial intelligence (AI) technologies in pharmacy practice, including but not limited to ChatGPT, large language models (LLMs), generative AI, machine learning, deep learning, natural language processing (NLP), AI-enabled robotic technologies, and AI-assisted clinical decision-support systems.	Studies investigating AI in general healthcare without a pharmacy practice focus; studies focusing on industrial pharmacy, pharmaceutical manufacturing, drug discovery, pharmaceutical sciences, or formulation development; technical AI or machine-learning algorithm development or validation studies; studies evaluating only AI-versus-pharmacist accuracy or performance without assessing AI implementation in pharmacy practice; studies focusing primarily on patient perceptions of AI or patient-directed AI interventions; and routine pharmacy automation or conventional rule-based clinical decision-support systems without an explicitly identified AI component.
Context (C)	Pharmacy practice in one or more Middle Eastern countries.	Studies conducted outside the Middle East.
Type of study	Original empirical research using quantitative, qualitative, or mixed-methods designs that addressed one or more review objectives.	Reviews, systematic reviews, scoping reviews, bibliometric studies, conference abstracts, conference proceedings, editorials, letters, commentaries, study protocols, corrigenda, errata, and abstracts without full text.
Language	English-language publications.	Publications in languages other than English.

### 2.4 Study selection

All search results were exported to EndNote 21 (Clarivate Analytics, Philadelphia, PA, USA), where duplicate records were identified and removed. The titles and abstracts of the remaining studies were independently screened by two reviewers (FA and FKA) according to the predefined eligibility criteria. Potentially eligible studies underwent full-text assessment independently by the same reviewers. Where full-text articles were unavailable, efforts were made to obtain them through institutional library services or by contacting the study authors. Studies that did not meet the inclusion criteria or for which the full text could not be obtained were excluded, and the reasons for exclusion were recorded. Disagreements regarding study eligibility were resolved through discussion until consensus was achieved. The study selection process is illustrated using the PRISMA-ScR flow diagram (Figure 1).

**Figure 1:**
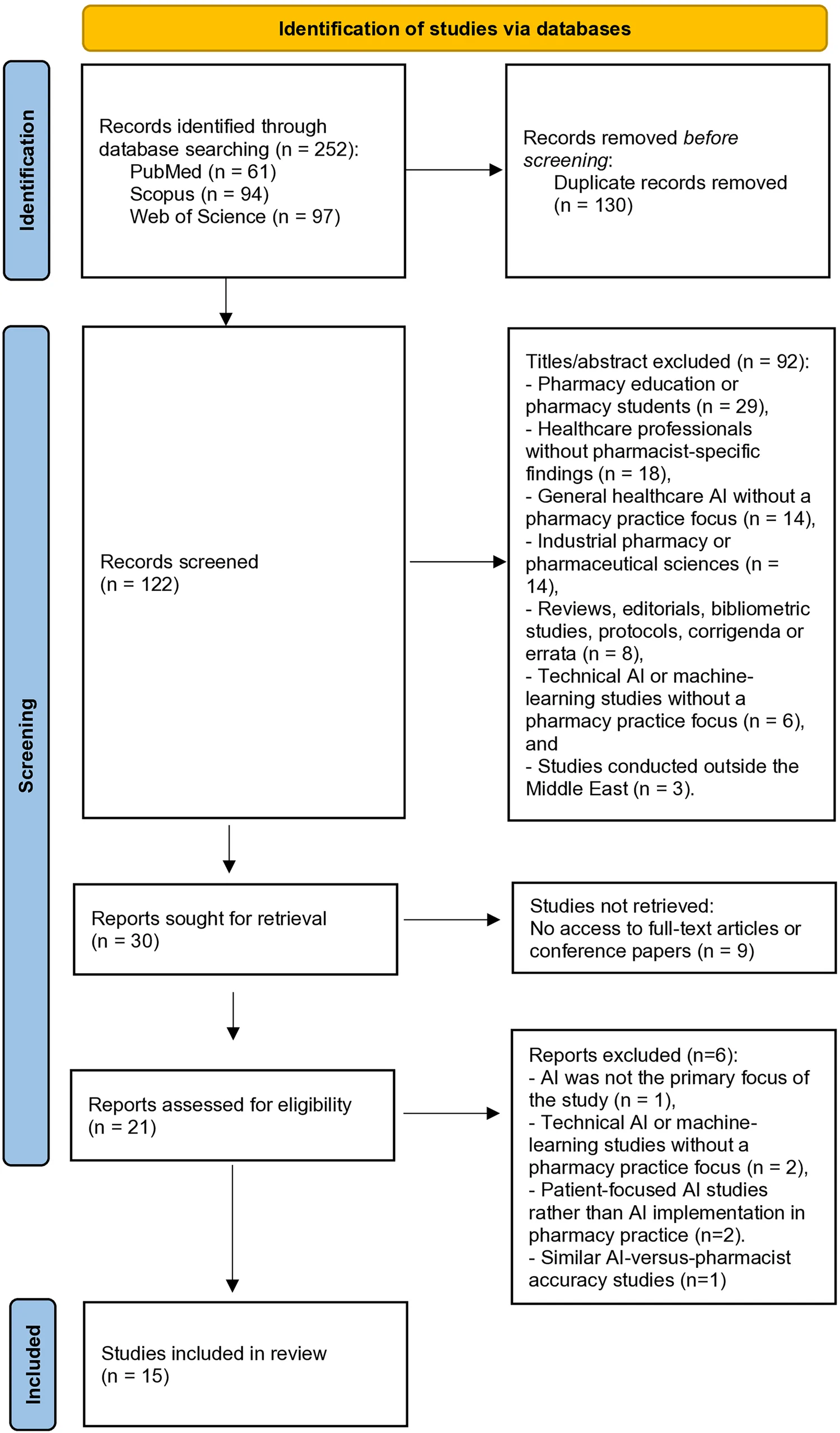
PRISMA-nscR flow diagram. The flow diagram outlines the study selection and screening process, including identification, screening, and inclusion stages.

### 2.5 Data extraction

Data from each included study were independently extracted by FA and FKA using a standardized data extraction form. Extracted information included author and year of publication, country, study design, pharmacy practice setting, participant characteristics and sample size, type of AI technology investigated, AI applications, current or intended utilization, pharmacists' awareness, knowledge, attitudes and perceptions, perceived or anticipated benefits, barriers and implementation challenges, and ethical or regulatory considerations. The extraction form was developed according to the objectives of the review and was piloted on a small number of included studies before full data extraction to ensure that relevant information was captured consistently. Any discrepancies in data extraction were resolved through discussion and consensus.

### 2.6 Data analysis and presentation

Extracted data were synthesized descriptively using narrative summary methods. Findings were grouped according to the objectives of the review, including:

- current AI applications in pharmacy practice;
- prevalence of AI use among pharmacists;
- pharmacists' awareness, knowledge, attitudes, perceptions, willingness, and acceptance of AI;
- reported benefits of AI implementation;
- barriers, facilitators, ethical considerations, and implementation challenges; and
- research gaps and future directions.

Where appropriate, findings were further organized according to AI technology (e.g., ChatGPT, large language models, machine learning, robotics, and clinical decision support systems) and pharmacy practice setting (community pharmacy, hospital pharmacy, and clinical pharmacy).

## 3 Results

### 3.1 Search results and study selection

The literature search identified 252 records across three electronic databases: PubMed (*n* = 61), Scopus (*n* = 94), and Web of Science (*n* = 97). Following the removal of 130 duplicate records, 122 unique records remained for title and abstract screening. Of these, 92 records were excluded because they did not meet the predefined eligibility criteria. The full texts of the remaining 30 articles were sought; however, 9 reports could not be retrieved because the full texts were unavailable or the publications were available only as conference papers. Consequently, 21 full-text articles were assessed for eligibility. Following full-text review, six studies were excluded because AI was not the primary focus (*n* = 1), they were technical AI or machine-learning studies without a pharmacy practice focus (*n* = 2), they focused on patient-directed AI rather than the implementation of AI in pharmacy practice (*n* = 2), or they evaluated the accuracy of AI by comparing AI-generated responses with pharmacists' responses rather than investigating AI implementation in pharmacy practice (*n* = 1). Ultimately, 15 studies met the eligibility criteria and were included in the scoping review (Figure 1). All included studies were published in English; therefore, no translation was required. Percentage agreement between the two reviewers on inclusion/exclusion decisions was 90% during both the title/abstract screening and full-text eligibility assessment. Percentage agreement was calculated as the number of records for which both reviewers reached the same decision divided by the total number of records assessed, multiplied by 100.

### 3.2 Characteristics of the included studies

The characteristics of the included studies are summarized in Table 3. A total of 15 studies published between 2023 and 2026 were included in this scoping review, reflecting the rapidly growing interest in the application of artificial intelligence (AI) in pharmacy practice across the Middle East (7–21).

**Table 3:** Characteristics of the included studies (*n* = 15).

Study	Country	Design	Pharmacy setting	Participants (n)	AI technology	Review objective(s) addressed
Abu-Farha et al., 2023 (7)	Jordan	Cross-sectional survey	Community pharmacy	221	ChatGPT	Perceptions and willingness to integrate ChatGPT
Jarab et al., 2023 (8)	Jordan	Cross-sectional survey	Community pharmacy	401	Artificial intelligence	Attitudes, willingness, and implementation barriers
Syed & Al-Rawi, 2024 (11)	Saudi Arabia	Cross-sectional survey	Community pharmacy	273	Artificial intelligence	Awareness, perceptions, and opinions
Jaber et al., 2024 (9)	Middle East [multinational; Lebanon had the highest representation (32.0%)]	Cross-sectional survey	Multiple pharmacy settings	328	Artificial intelligence	Knowledge, attitudes, and practices
Jairoun et al., 2024 (10)	United Arab Emirates	Qualitative study	Community pharmacy	35	ChatGPT	Benefits, risks, and applications
Hasan et al., 2024 (16)	Middle East (multinational: Jordan, Lebanon, Egypt, Palestine, Saudi Arabia, and Kuwait)	Cross-sectional survey	Multiple pharmacy settings	501	Artificial intelligence	Ethical considerations
Taha et al., 2024 (13)	Egypt	Cross-sectional survey	Multiple pharmacy settings	428	ChatGPT	Perceptions, concerns, and practice
Alghitran et al., 2025 (20)	Saudi Arabia	Cross-sectional survey	Multiple pharmacy settings	1022	General AI and ChatGPT	Knowledge, attitudes, and integration
Jarab et al., 2025 (15)	United Arab Emirates	Cross-sectional survey	Community pharmacy	430	AI-driven robotic tools	Utilization, benefits, and concerns
Shabana et al., 2025 (18)	Qatar	Cross-sectional survey	Hospital pharmacy	93	Artificial intelligence	Attitudes, willingness, and barriers
Said et al., 2026 (14)	United Arab Emirates	Cross-sectional survey	Multiple pharmacy settings	340	Artificial intelligence	Perceptions and implementation concerns
Jarab et al., 2026 (12)	United Arab Emirates	Cross-sectional survey	Community pharmacy	528	Large language models (LLMs)	Benefits, concerns, and implementation barriers
Kahwaji et al., 2026 (19)	Syria	Cross-sectional survey	Multiple pharmacy settings	400	Generative AI	Adoption and knowledge
Khaled et al., 2026 (17)	Saudi Arabia	Cross-sectional survey	Community pharmacy	117	Artificial intelligence	Attitudes toward AI integration
Alrohaimi et al., 2026 (21)	Saudi Arabia	Cross-sectional survey	Multiple pharmacy settings	3000	Artificial intelligence	Impact and implementation challenges

The included studies were conducted across multiple Middle Eastern countries or regions. Saudi Arabia contributed the largest number of country-specific studies (*n* = 4) (11, 17, 20, 21), followed by the United Arab Emirates (*n* = 4) (10, 12, 14, 15) and Jordan (*n* = 2) (7, 8), while Egypt (13), Qatar (18), and Syria (19) each contributed one country-specific study. Two additional studies were multinational Middle Eastern studies (9, 16). No country-specific eligible studies were identified from Bahrain, Kuwait, Lebanon, Oman, Iraq, Israel, Palestine, Yemen, Turkiye, or Iran. However, Saudi Arabia, Kuwait, Jordan, Lebanon, Egypt, and Palestine were represented within a multinational study (16), with Lebanon also represented in the multinational study by Jaber et al. (9).

The vast majority of studies employed a cross-sectional survey design (*n* = 14, 93.3%) (7–9, 11–21), whereas only one study (6.7%) used a qualitative methodology to explore pharmacists' perspectives on ChatGPT (10). Sample sizes varied substantially, ranging from 35 to 3,000 participants, with a total of 8,117 participants across all included studies (7–21).

Regarding pharmacy settings, multiple pharmacy settings were the most frequently investigated (*n* = 7) (9, 13, 14, 16, 19–21), followed by community pharmacies (*n* = 7) (7, 8, 10–12, 15, 17), while one study was conducted exclusively in hospital pharmacies (18).

The AI technologies investigated included artificial intelligence in general (*n* = 8) (8, 9, 11, 14, 16, 18, 20, 21), ChatGPT (*n* = 4) (7, 10, 13, 20), AI-enabled robotic technologies (*n* = 1) (15), large language models (LLMs) (*n* = 1) (12), and generative AI (*n* = 1) (19). These technology categories were not necessarily mutually exclusive, as some studies examined AI broadly while also assessing specific technologies such as ChatGPT. Where this occurred, studies were classified according to all relevant technology categories rather than being assigned exclusively to a single category. Most studies explored pharmacists' knowledge, awareness, attitudes, perceptions, willingness to adopt AI, and implementation barriers (7–18, 20, 21), whereas fewer studies examined AI utilization (9, 15), ethical considerations (16), AI-enabled robotic technologies (15), adoption of generative AI (19), or implementation challenges (21).

Overall, the available evidence indicates that research on AI in pharmacy practice within the Middle East is recent and predominantly survey-based, with a primary focus on pharmacists' perceptions and readiness to adopt AI technologies rather than evaluating their implementation or clinical effectiveness (7–21).

Table 4 presents a comprehensive mapping of the included studies according to the review objectives, summarizing the AI applications investigated, AI utilization, pharmacists' awareness, knowledge, attitudes, and perceptions, perceived benefits, implementation barriers, and ethical and regulatory considerations. The findings presented in Table 4 provide the basis for the thematic synthesis described in the following sections.

**Table 4:** Mapping of included studies and key findings by the review objectives.

Study	AI applications investigated or proposed	Current utilization/Intended use	Perceptions, awareness, knowledge, or attitudes	Barriers and implementation challenges	Perceived or anticipated benefits	Ethical or regulatory considerations
Abu-Farha et al., 2023 (7)	ChatGPT for medication information and clinical decision support	48.4% were willing to integrate ChatGPT	47.5% high perceived benefit; 37.0% high concern	Limited human judgment; inability to understand complex situations; reliability and accuracy concerns	Faster access to drug information; support for pharmacy tasks	>70% believed ChatGPT cannot replace human ethical judgment
Jarab et al., 2023 (8)	General AI in community pharmacy	50.9% were willing to adopt AI	50.4% showed positive attitudes; 82.0% wanted AI updates; 79.5% desired AI training	Lack of software/hardware (79.2%); need for human supervision (76.4%); high cost (74.6%)	Improved efficiency and time saving	Human supervision was considered essential
Syed & Al-Rawi, 2024 (11)	AI and robotics for dispensing, medication safety, and decision support	Not assessed	94.5% were aware of AI; 63.4% viewed AI as supportive; 25.6% believed AI may replace professionals	Job displacement (12.8%)	Perceived reduction in medication errors (84.0%); perceived improvement in healthcare access (86.0%); perceived improvement in decision-making (87.9%)	Workforce displacement
Jaber et al., 2024 (9)	General AI	57.3% demonstrated adequate AI practice	44.8% moderate AI knowledge; 49.1% positive attitudes. AI knowledge, attitudes, and practice significantly associated (*p* < 0.001); cross-sectional association only	Knowledge gaps, limited preparation, and the need to integrate AI effectively into pharmacy workflows	Perceived improvement in workflow and medication management	Not assessed
Jairoun et al., 2024 (10)	ChatGPT for counseling, adherence, dispensing, and medication management	Not assessed	Positive perceptions toward ChatGPT for counseling and medication management	Outdated information; inaccurate recommendations; limited medication details	Improved medication adherence, counseling, medication safety, medication management, reduced errors, and streamlined dispensing	Need for evidence-based use, professional verification, and protection of patient safety
Hasan et al., 2024 (16)	General AI	Not assessed	Considerable ethical concern regarding AI implementation	Lack of legal regulation (67.0%); job displacement (62.9%); privacy (58.9%); cybersecurity (58.9%)	Not assessed	Privacy, autonomy, transparency, justice, governance
Taha et al., 2024 (13)	ChatGPT for drug information, ADR management, patient education	70.6% used ChatGPT; drug information (30.6%); ADR management (35.5%); dosage regimen determination (25.9%), and medication reconciliation (7.0%)	73.6% recognized benefits; 73.6% recommended ChatGPT	Inaccurate responses (67.1%); privacy (61.7%); cybersecurity (59.8%); algorithmic bias (53.5%)	Drug-information retrieval, patient education, workflow optimization, and support for medication-related tasks	Accuracy, privacy, bias, and the need for responsible implementation and human oversight
Alghitran et al., 2025 (20)	ChatGPT for clinical decisions, counseling, and medication management	78.3% used AI; ChatGPT moderate/high use (∼67%); pharmaceutical inquiries (66%); collaborative decisions (62%)	78.7% familiar with AI; 90.1% recognized ChatGPT; 64.1% positive attitudes	No formal AI training (92.0%); ethical concerns (64.0%)	Improved workflow (61.0%); improved quality of care (79.0%)	Need for governance and structured AI training
Jarab et al., 2025 (15)	AI-driven robotics for inventory, dispensing, packaging, and labeling	53.5% familiar with robotics; 21.2% trained; 20.0% regular users; inventory management (39.5%); automated dispensing (36.0%)	Benefits: reduced technical workload (41.4%); improved workflow (30.7%); reduced dispensing errors (29.7%). Median concern score: 9/14	Major concerns: system malfunction (59.8%); patient preference for human pharmacists (58.0%); reduced pharmacist–patient interaction (55.8%); implementation cost (55.5%); lack of training (55.1%)	Applications: inventory management (60.0%); public health (46.7%); prescription refills (45.6%); home delivery (44.0%); medication review (42.3%)	Patient privacy and data security, cybersecurity, controlled-substance security, and legal/regulatory compliance
Shabana et al., 2025 (18)	AI for hospital pharmacy, adherence, and medication management	Pharmacists reported a favorable median willingness of 50; a median attitude of 53	Not assessed	Inadequate AI training (75.3%); inability to address patients' emotions (75.3%)	Perceived improvement in medication adherence (79.6%)	Loss of empathy
Said et al., 2026 (14)	AI for rapid data analysis, workflow, and medication safety	Not assessed	Positive perceptions toward AI	Cybersecurity (4.2/5); privacy (4.1/5); job displacement (3.9/5)	Multitasking/data analysis (4.1/5); service quality (3.9/5); patient follow-up (3.8/5)	Privacy, cybersecurity, workforce displacement, preservation of human care, and the need for regulatory frameworks
Jarab et al., 2026 (12)	LLMs for personalized care, information support and 24-hour assistance	Not assessed	Potential benefits acknowledged, but concerns remained high	Technical failure (97.5%); cybersecurity (97.2%); limited empathy (95.6%); human supervision (54.7%); insufficient training (32.4%)	Perceived improvement in patient outcomes (77.0%); personalized care (74.4%); round-the-clock support (37.3%)	Cybersecurity, privacy, limited empathy, cultural sensitivity, ethical reasoning, human supervision, and governance
Kahwaji et al., 2026 (19)	Generative AI and ChatGPT	52.9% used ChatGPT; 55% used AI daily; 50% used AI in pharmacy practice	64.0% were familiar with AI; 77.5% supported AI applications for pharmacy; 66.2% believed AI supports clinical decisions; 53.0% believed AI would not replace pharmacists	Internet restrictions, limited access to AI platforms, language limitations, insufficient training, and absence of specific national regulation	Perceived improvements in workflow efficiency, medication safety, and clinical decision-making	Need for regulation, structured training, transparency, human oversight, and context-appropriate governance
Khaled et al., 2026 (17)	AI for dispensing, medication safety, and drug-related problem identification	Not assessed	81.2% were aware of AI; 51.6% had sufficient knowledge; 58.9% did not believe AI would replace pharmacists	Limited AI knowledge; infrastructure limitations	Perceived reduction in medication errors (71.6%)	Concern regarding replacement of pharmacists; emphasis on AI complementing professional roles
Alrohaimi et al., 2026 (21)	AI for diagnosis, prediction, medication management, and decision support	Not assessed	General support for AI as a decision-support tool	Data privacy, cybersecurity, algorithmic bias, lack of transparency, and over-reliance on automated systems	Perceived improvements in diagnostic accuracy, treatment prediction, medication management, and workflow efficiency	Patient consent, algorithmic fairness, explainability, professional accountability, human oversight, and robust governance frameworks

### 3.3 AI applications in pharmacy practice

The included studies identified a range of actual, reported, and potential AI applications across both clinical and operational aspects of pharmacy practice. ChatGPT and other large language models (LLMs) were primarily investigated as tools to support medication information retrieval, medication counselling, patient education, medication management, and medication adherence support, with pharmacists also recognizing their potential to assist clinical decision-making during pharmaceutical care (7, 10, 12, 13, 20). However, only one study specifically evaluated pharmacists' perspectives on LLM implementation (12), whereas the remaining studies focused predominantly on ChatGPT (7, 10, 13, 20).

Broader AI technologies were investigated or proposed for clinical decision support, medication safety, dispensing support, workflow optimization, identification of drug-related problems, and pharmaceutical care, with several studies also reporting pharmacists' perceptions that these technologies could improve decision-making and reduce medication-related errors (8, 9, 11, 14, 17, 18, 21). AI-enabled robotic technologies represented a distinct application area and were evaluated primarily for inventory management, automated dispensing, robotic packaging, robotic labeling, prescription refills, medication review, and home medication delivery within community pharmacy practice (15).

Overall, the included studies suggest that AI has the potential to support multiple stages of the medication-use process, ranging from medication information provision and clinical decision support to dispensing and pharmacy operations. Nevertheless, the available evidence was largely based on pharmacists' perceptions of potential applications, with relatively few studies evaluating actual implementation or real-world clinical use (9, 13, 15, 19, 20).

The technologies identified in the included studies differed substantially in their functions and implementation requirements.

ChatGPT, LLMs, and generative AI were predominantly used or proposed as information- and communication-support tools, including medication information retrieval, patient counselling, education, medication management, and support for clinical decision-making (7, 10, 12, 13, 19, 20). In contrast, AI-enabled robotic technologies were primarily operational tools supporting dispensing, packaging, labelling, inventory management, prescription refills, and medication delivery (15). Studies investigating general AI described a broader range of potential applications, particularly clinical decision support, medication safety, identification of drug-related problems, and workflow optimization (8, 9, 11, 14, 17, 18, 21), although the specific underlying AI technology was not always clearly defined. These categories were therefore considered separately when interpreting utilization, benefits, and barriers to implementation. To facilitate comparison across the different AI technologies identified in the included studies, their principal pharmacy applications, apparent adoption patterns, and key implementation considerations are summarized in Table 5.

**Table 5:** Technology-specific synthesis of AI applications, adoption patterns, and implementation considerations in Middle Eastern pharmacy practice.

Technology category	Main pharmacy applications	Apparent adoption pattern	Key implementation requirements/barriers
ChatGPT, LLMs, and generative AI	Medication information, patient counselling, education, medication management, and decision support	Most visible and accessible technologies; evidence of individual pharmacist use	Accuracy, reliability, privacy, training, and professional oversight
General AI and AI-assisted clinical decision support	Medication safety, clinical decision support, identification of drug-related problems, and workflow optimization	Evidence predominantly relates to perceptions, awareness, and readiness; limited routine implementation	System integration, infrastructure, training, validation, governance, and accountability
AI-enabled robotics	Dispensing, packaging, labelling, inventory management, prescription refills, and medication delivery	Awareness exceeds routine use; relatively limited implementation	Equipment and infrastructure requirements, workflow integration, staff training, and organizational investment

### 3.4 AI utilization among pharmacists

To avoid conflating different stages of AI adoption, the terminology used in this review was interpreted as follows: awareness refers to familiarity with or knowledge of an AI technology; willingness refers to an expressed intention or readiness to use or adopt AI; individual use refers to self-reported previous or occasional use of an AI tool by a pharmacist; routine use refers to regular or recurring use of AI as part of pharmacy-related practice; and organization-level implementation refers to formal integration of an AI technology into pharmacy services, workflows, or institutional systems. Where the included studies did not clearly distinguish between these levels, findings were reported using the terminology of the original study and were not interpreted as evidence of routine or organizational implementation.

Only a small proportion of the included studies examined actual AI utilization in pharmacy practice, whereas most focused on pharmacists' awareness, knowledge, attitudes, or willingness to adopt AI technologies (7–9, 11, 12, 14, 16–18, 21). Among studies reporting AI use, ChatGPT was the most frequently used AI application. In Egypt, 70.6% of pharmacists reported previous use of ChatGPT (13), while a national survey from Saudi Arabia found that 78.3% of pharmacists had used AI tools at least once in their professional practice (20). Similarly, a multinational survey reported that 57.3% of pharmacists demonstrated adequate AI practice, although this reflected self-reported AI-related practice rather than routine implementation in pharmacy services (9).

Evidence regarding routine implementation of AI technologies in pharmacy practice remained limited. In Syria, although more than half of pharmacists reported using generative AI, adoption was largely recent and knowledge levels varied considerably, reflecting an early stage of implementation (19). Likewise, the only study evaluating AI-enabled robotic technologies reported that, despite 53.5% of community pharmacists being familiar with robotic systems, only 20.0% reported regular use and 21.2% had received formal robotics training (15). Collectively, these findings indicate that while awareness and initial exposure to AI technologies are increasing across the Middle East, their routine integration into everyday pharmacy practice remains limited (9, 13, 15, 19, 20).

### 3.5 Pharmacists' awareness, knowledge, attitudes, and perceptions toward AI

Overall, the available evidence indicates that pharmacists across the Middle East demonstrate high awareness and generally positive attitudes toward AI technologies, although knowledge and readiness for implementation vary between studies. High levels of AI awareness were consistently reported, with 94.5% of community pharmacists in Saudi Arabia indicating awareness of AI technologies (11), while other studies similarly reported widespread familiarity with AI and ChatGPT among pharmacists (9, 13, 17, 20).

Pharmacists generally perceived AI as a supportive technology capable of enhancing, rather than replacing, professional pharmacy practice. Positive attitudes toward AI implementation were reported across community, hospital, and mixed pharmacy settings, with many pharmacists recognizing AI's potential to improve clinical decision-making, medication safety, workflow efficiency, and pharmaceutical care (8, 9, 11, 14, 17, 18, 20, 21). Nevertheless, concerns regarding workforce displacement remained evident. For example, 25.6% of pharmacists in Saudi Arabia believed that AI could eventually replace healthcare professionals (11), while concerns regarding job displacement were also identified in multinational and hospital-based studies (16, 18).

Willingness to adopt AI was likewise consistently reported. Approximately 48.4% of community pharmacists in Jordan expressed willingness to integrate ChatGPT into pharmacy practice (7), while 50.9% reported willingness to adopt AI in general (8). Furthermore, pharmacists demonstrated a strong interest in AI education, with 82.0% requesting regular AI updates and 79.5% expressing a desire for formal AI training (8), highlighting a recognized need to strengthen AI competencies before widespread implementation.

Although perceptions toward AI were generally favorable, pharmacists' knowledge was less consistent across studies. A multinational survey found that only 44.8% of pharmacists demonstrated moderate AI knowledge despite reporting positive attitudes and AI-related practice (9), suggesting that favorable perceptions do not necessarily translate into adequate AI competency. Qualitative evidence further showed that pharmacists viewed ChatGPT as a valuable adjunct for medication counselling, medication management, patient education, medication adherence support, and dispensing workflows, while emphasizing that pharmacist oversight remains essential to ensure safe and appropriate clinical use (10).

Overall, the findings indicate that pharmacists are receptive to AI adoption; however, successful implementation will require continued professional education, competency development, and organizational support to translate positive attitudes into effective clinical practice (8, 9, 17, 18, 20, 21).

### 3.6 Perceived benefits of AI in pharmacy practice

Across the included studies, pharmacists consistently perceived AI as having the potential to enhance both clinical practice and pharmacy operations. The most frequently reported benefits included improved clinical decision-making, enhanced medication safety, faster access to drug and medication information, improved workflow efficiency, and greater operational efficiency (7, 9–15, 17, 18, 20, 21).

Several studies reported that pharmacists perceived AI as a valuable clinical support tool capable of facilitating medication-related decision-making and improving the quality of pharmaceutical care. ChatGPT and other large language models were considered particularly useful for providing medication information, supporting medication counselling, assisting patient education, and facilitating medication management during routine pharmacy practice (7, 10, 12, 13, 20). Community pharmacists also recognized the potential of AI to improve medication adherence and enhance communication with patients (10, 13).

Studies evaluating general AI technologies similarly reported perceived benefits in medication safety, decision support, and workflow optimization. In Saudi Arabia, most pharmacists believed that AI could improve clinical decision-making (87.9%), increase access to healthcare services (86.0%), and reduce medication errors (84.0%) (11). Likewise, pharmacists from multinational and regional studies reported that AI could improve workflow efficiency, facilitate medication management, and enhance pharmaceutical care (9, 14, 17, 18, 21).

The only study evaluating AI-enabled robotic technologies reported operational rather than clinical benefits. Community pharmacists perceived robotic systems as reducing technical workload (41.4%), improving workflow efficiency (30.7%), reducing dispensing errors (29.7%), reducing prescribing errors (27.9%), and improving patient satisfaction (23.7%) (15). These findings suggest that robotic technologies are primarily viewed as tools for improving dispensing processes and pharmacy operations.

Overall, the available evidence indicates that pharmacists perceive AI primarily as a decision-support and workflow-enhancement technology that complements professional practice rather than replacing pharmacists. However, nearly all reported benefits were based on pharmacists' perceptions or anticipated advantages rather than evaluations of real-world clinical or operational outcomes (7–21).

### 3.7 Barriers to AI implementation

Despite generally positive attitudes toward AI, the included studies consistently identified multiple barriers that may hinder its implementation in pharmacy practice. The most frequently reported barriers were related to insufficient AI knowledge and training, with pharmacists across several countries expressing a need for formal education and professional development before AI could be safely integrated into routine practice (7–9, 12, 17, 18, 21).

Technical and organizational barriers were also commonly reported. Pharmacists identified limited technological infrastructure, inadequate availability of AI software and digital resources, implementation costs, lack of organizational readiness, and insufficient institutional support as major obstacles to AI adoption (8, 9, 12, 14, 17, 18, 21). The study evaluating AI-enabled robotic technologies likewise found that limited training and infrastructure represented important challenges to wider implementation in community pharmacies (15).

Concerns regarding the reliability, transparency, and safe use of AI-generated recommendations were consistently highlighted. Pharmacists expressed reservations about the accuracy of AI outputs, algorithmic bias, limited explainability, cybersecurity threats, data privacy, and the potential for AI-generated misinformation or clinical errors if used without professional oversight (7, 10, 12, 13, 16, 17, 21). Ethical and regulatory concerns, including accountability for AI-assisted decisions and the absence of clear governance frameworks, were also frequently identified (12, 16, 17, 21).

Several studies additionally highlighted concerns regarding the impact of AI on professional practice. Pharmacists reported that excessive reliance on AI could reduce critical thinking, diminish pharmacist–patient interaction and empathy, and increase dependence on automated recommendations (10, 16, 18). Concerns regarding potential workforce displacement were also reported, although most pharmacists viewed AI as a complementary tool intended to support, rather than replace, professional judgment (11, 16, 18).

Overall, the findings suggest that successful AI implementation in pharmacy practice will require coordinated investment in education and workforce training, digital infrastructure, organizational readiness, and robust ethical and regulatory frameworks to ensure the safe, effective, and responsible use of AI technologies (8, 9, 12, 16–18, 21).

### 3.8 Ethical and regulatory considerations

Ethical and regulatory considerations emerged as a recurring theme across the included studies, particularly those evaluating generative AI and broader AI implementation in pharmacy practice. Frequently reported concerns included patient privacy, confidentiality of health information, cybersecurity, algorithmic bias, transparency, explainability of AI-generated recommendations, and accountability for AI-assisted clinical decisions (7, 10, 12, 16, 17, 21).

Several studies emphasized that AI should function as a clinical decision-support tool that complements, rather than replaces, pharmacists' professional judgment. Pharmacists consistently highlighted the importance of maintaining human oversight to verify AI-generated recommendations, particularly for medication-related decisions and patient counselling, to minimize the risk of clinical errors and inappropriate recommendations (7, 10, 12, 17, 21).

Legal and regulatory uncertainty was also identified as a major barrier to implementation. Authors highlighted the absence of comprehensive governance frameworks, standardized regulations, and clearly defined professional responsibilities for AI-assisted care, emphasizing the need for policies that address accountability, data governance, validation of AI systems, and ethical deployment within pharmacy practice (12, 16, 17, 21).

Across the included studies, authors consistently recommended strengthening AI governance, establishing clear legal and regulatory frameworks, integrating AI ethics and digital health competencies into pharmacy education, and maintaining ongoing professional oversight to support the safe, ethical, and responsible implementation of AI in pharmacy practice (8, 12, 16, 17, 21).

## 4 Discussion

To our knowledge, based on searches of PubMed, Scopus, and Web of Science and a literature check of relevant reviews, this is the first scoping review to comprehensively synthesize evidence on artificial intelligence (AI) in pharmacy practice across the Middle East. Fifteen studies published between 2023 and 2026 were identified, demonstrating rapidly increasing research interest in this field. However, despite growing enthusiasm for AI, the available evidence remains at an early stage of development. Most studies were cross-sectional surveys evaluating pharmacists' awareness, perceptions, and willingness to adopt AI technologies, whereas only a small number examined actual AI utilization and none evaluated clinical effectiveness, implementation outcomes, patient outcomes, or economic impact (7–21). This contrast between widespread professional interest and limited implementation represents the principal finding of this review and highlights the gap between technological readiness and practical integration within pharmacy services.

One notable finding was the rapid emergence of generative AI, particularly ChatGPT and large language models (LLMs), as the dominant AI technologies investigated in pharmacy practice. Earlier reviews of AI in pharmacy largely focused on machine learning, clinical decision-support systems, and robotics (3–5, 25), whereas the studies included in this review demonstrate a clear shift toward generative AI applications for medication information, patient counselling, medication management, and pharmaceutical decision support (7, 10, 12, 13, 20). This likely reflects the widespread public availability of ChatGPT since late 2022. Among the technologies identified in this review, ChatGPT and other generative AI/LLM tools therefore appear to represent the current forerunners in pharmacist-level adoption. Their relative accessibility may be partly explained by their ease of individual access and use for information-based tasks, with fewer immediate requirements for specialized physical infrastructure or major workflow redesign than technologies such as robotic dispensing. Nevertheless, their apparent accessibility should not be interpreted as readiness for unsupervised clinical use, because concerns regarding accuracy, privacy, accountability, and the need for professional oversight remain (3–5, 25).

In contrast, AI-enabled robotic technologies represent a more infrastructure-intensive form of AI implementation. Although 53.5% of community pharmacists in the UAE were familiar with robotic systems, only 20.0% reported regular use and 21.2% had received formal robotics training (15). Their implementation requires investment in equipment and technological infrastructure, integration into dispensing and operational workflows, staff training, and organizational support. AI-assisted clinical decision-support applications occupy a different position, as their adoption may depend on integration with institutional information systems and clinical data, together with validation, governance, and clearly defined accountability for AI-assisted decisions. Thus, adoption should not be viewed as uniform across AI technologies: relatively accessible generative AI tools may diffuse more rapidly at the individual pharmacist level, whereas robotics and integrated clinical systems are more dependent on organizational capacity and institutional investment.

As an interpretation of the available evidence, adoption may also be shaped by the context in which AI is implemented. Differences in organizational size and resources, workforce capacity, patient volume, digital infrastructure, institutional investment, access to AI technologies, and regulatory maturity may influence whether a technology can move from individual experimentation to routine pharmacy service delivery. At the national level, differences in digital-health strategies may further contribute to variation in exposure and implementation across countries (22–24). However, the included studies did not systematically compare adoption according to pharmacy size, staffing levels, patient volume, or organizational resources. Consequently, the present evidence does not permit firm conclusions regarding the independent contribution of these factors, highlighting an important priority for future implementation research.

Importantly, awareness, willingness, individual use, and implementation represent distinct stages of AI adoption and should not be interpreted interchangeably. High awareness or willingness indicates professional familiarity or receptiveness, while previous or occasional individual use demonstrates exposure rather than sustained integration into pharmacy services. Routine use requires recurring incorporation into pharmacy-related practice, whereas organization-level implementation implies formal integration into institutional workflows, systems, or service delivery. Viewed through this distinction, the evidence identified in this review predominantly reflects awareness, willingness, and individual experimentation, with substantially less evidence of routine use and very limited evidence of formal organization-level implementation. Moreover, because these constructs were operationalized using different questions and instruments across studies, the reported percentages should not be interpreted as directly comparable measures of AI adoption. Although pharmacists generally demonstrated high awareness of AI and expressed positive attitudes toward its adoption, this enthusiasm was not consistently accompanied by adequate knowledge or routine implementation (7–21). Several studies reported high awareness and favorable perceptions, yet comparatively fewer pharmacists demonstrated sufficient AI knowledge or regularly incorporated AI into practice (9, 13, 15, 19, 20). This discrepancy reflects a phenomenon increasingly reported across healthcare professions, whereby awareness develops more rapidly than competency (1, 2). Similar findings have been observed among physicians, nurses, and other healthcare professionals internationally, where confidence in AI frequently exceeds formal education and practical experience (1, 2). These findings suggest that positive attitudes alone are unlikely to result in successful implementation unless accompanied by structured competency development.

Consistent across nearly all studies was the perception that AI could enhance both clinical and operational aspects of pharmacy practice. Pharmacists perceived AI as improving medication information retrieval, clinical decision support, workflow efficiency, medication safety, and pharmaceutical care while reducing repetitive administrative activities (7, 9–15, 17, 18, 20, 21). These findings align with international systematic reviews demonstrating that AI has considerable potential to improve medication-related decision-making and pharmacy workflow (3–5, 25). However, an important distinction should be recognized: almost all reported benefits identified in this review were based on pharmacists' expectations or perceived advantages rather than objectively measured clinical outcomes (7–21). Consequently, although AI appears promising, robust evidence demonstrating improvements in medication safety, patient outcomes, healthcare quality, or efficiency within routine pharmacy practice in the Middle East remains scarce.

Implementation barriers were remarkably consistent across countries and pharmacy settings. Pharmacists repeatedly identified inadequate AI education, insufficient technological infrastructure, implementation costs, organizational readiness, and concerns regarding the reliability of AI-generated recommendations as major obstacles to adoption (7–10, 12–18, 21). These findings are consistent with implementation science frameworks, which emphasize that successful digital-health implementation depends not only on technology availability but also on organizational capacity, workforce preparedness, leadership support, and governance (1, 2). Importantly, many of the identified barriers are modifiable, suggesting that implementation challenges are unlikely to be overcome solely through technological advances and instead require coordinated educational, organizational, and policy interventions.

Ethical and regulatory issues also emerged as central considerations. Pharmacists consistently emphasized concerns regarding data privacy, cybersecurity, transparency, algorithmic bias, legal accountability, and professional responsibility when AI is incorporated into medication-related decision-making (7, 10, 12, 16, 17, 21). Rather than rejecting AI, pharmacists generally advocated for human oversight and viewed AI as a clinical decision-support tool that should complement—not replace—professional judgement (7, 10, 12, 17, 21). This perspective closely aligns with the World Health Organization's principles for trustworthy AI and reflects a growing international consensus that human accountability should remain central to AI-enabled healthcare (1).

### 4.1 Implication to practice and policy

The findings of this review have several practical and policy implications. First, pharmacy schools should incorporate AI literacy, prompt engineering, digital health, AI ethics, and critical appraisal of AI-generated information into undergraduate and postgraduate pharmacy curricula. Second, healthcare organizations should provide structured continuing professional development programmes to improve pharmacists' competencies before large-scale AI implementation. Third, healthcare institutions should adopt governance frameworks defining acceptable AI use, human oversight requirements, validation procedures, cybersecurity standards, and accountability mechanisms for AI-assisted clinical decisions. Finally, policymakers should develop national regulatory frameworks that support innovation while ensuring patient safety, transparency, equity, and responsible AI deployment. Given the rapid expansion of national digital-health initiatives across several Middle Eastern countries, integrating pharmacy-specific AI strategies within broader national digital transformation programmes may accelerate safe implementation while maintaining professional standards.

### 4.2 Study strengths and limitations

This review has several strengths. To our knowledge, based on searches of PubMed, Scopus, and Web of Science and a literature check of relevant reviews, this is the first scoping review to comprehensively synthesize evidence on AI in pharmacy practice across the Middle East. The review followed established Joanna Briggs Institute methodology and PRISMA-ScR reporting guidance and included multiple AI technologies, pharmacy settings, and countries, thereby providing a broad overview of current evidence.

Nevertheless, several limitations should be acknowledged. First, the available evidence was limited to 15 studies and was heavily dominated by cross-sectional surveys, with only one qualitative study and no implementation, longitudinal, or interventional research. Accordingly, statistical associations reported in the included cross-sectional studies should not be interpreted as evidence of temporal, directional, or causal relationships between AI knowledge, attitudes, perceptions, and use. Second, the available evidence was concentrated in a limited number of Middle Eastern countries, leaving substantial geographic gaps across the region. Third, most outcomes were based on self-reported perceptions rather than objectively measured AI use or clinical outcomes, introducing the potential for response and social desirability bias. Fourth, the rapidly evolving nature of AI means that new evidence is likely to emerge quickly, particularly regarding generative AI and large language models. Fifth, the literature search was limited to PubMed, Scopus, and Web of Science, supplemented by manual screening of the reference lists of included studies. Although these databases provide broad biomedical and multidisciplinary coverage, Embase and regional databases such as IMEMR/WHO EMRO were not searched, and the review did not include a dedicated grey literature search. Consequently, relevant evidence from non-indexed regional journals, conference proceedings, government reports, institutional documents, or other grey literature may have been missed. This limitation may be particularly relevant in the rapidly evolving field of AI, where implementation and policy developments may precede publication in peer-reviewed journals. Sixth, the review was restricted to English-language publications, which introduces the possibility of language bias. Although English is widely used for scientific publication in medicine, pharmacy, and health sciences across the Middle East, relevant studies published in Arabic or other regional languages, particularly in local or non-indexed journals, may not have been captured. Therefore, the absence of eligible studies from some Middle Eastern countries should be interpreted as an absence of evidence identified within the scope and search strategy of the present review rather than as evidence that AI or digital-health research is absent from these countries more broadly, particularly as this review specifically required studies to examine AI within pharmacy practice and meet the predefined eligibility criteria.

An additional limitation is the substantial heterogeneity in outcome definitions and measurement approaches across the included studies.

Studies assessed different constructs, including awareness, knowledge, attitudes, willingness to adopt AI, previous use, and routine use, using different survey items, response scales, and study-specific instruments. Consequently, reported percentages across studies are not directly comparable; for example, a high proportion reporting awareness of AI should not be interpreted as equivalent to the proportion reporting previous or routine AI use. The percentages summarized in this review should therefore be interpreted descriptively within the context of each individual study rather than as directly comparable estimates or evidence of regional trends. The lack of standardized and validated measures also limits cross-study synthesis and highlights the need for consistent instruments in future research. Finally, the classification of AI technologies was not always mutually exclusive because some primary studies evaluated AI broadly while simultaneously examining specific tools such as ChatGPT. In addition, several studies used the term ‘AI’ without clearly specifying the underlying technology. Although technologies were categorized according to the descriptions provided in the original studies, some overlap between general AI, generative AI, ChatGPT, and LLM categories was therefore unavoidable.

### 4.3 Suggestion for future work

Future research should move beyond descriptive surveys toward implementation and effectiveness research. Prospective implementation studies, pragmatic clinical trials, and mixed-methods evaluations are needed to determine whether AI improves medication safety, pharmacist productivity, workflow efficiency, patient outcomes, and healthcare costs in real-world pharmacy practice. Standardized instruments for assessing AI knowledge, competency, and readiness would facilitate comparisons across countries. Comparative studies involving multiple Middle Eastern healthcare systems are also needed to understand how differences in regulation, infrastructure, and digital-health maturity influence AI adoption. Finally, future research should evaluate the effectiveness of AI education programmes, governance frameworks, and implementation strategies, as well as examine patient perspectives, ethical outcomes, and economic evaluations of AI integration into pharmacy services.

## 5 Conclusions

Artificial intelligence is rapidly emerging as an important component of pharmacy practice across the Middle East, with pharmacists demonstrating generally positive attitudes toward its adoption and recognizing its potential to enhance clinical decision-making, medication safety, and pharmacy workflow. However, current evidence indicates that AI implementation remains in its early stages, with the literature predominantly reflecting pharmacists' perceptions of anticipated benefits rather than evidence of demonstrated clinical effectiveness. Educational deficiencies, technological infrastructure, governance, and ethical concerns remain major barriers to implementation. Future efforts should focus on strengthening AI competencies, developing robust regulatory frameworks, and generating high-quality implementation evidence to ensure that AI is integrated safely, effectively, and responsibly into pharmacy practice.

## Data Availability

The original contributions presented in the study are included in the article, further inquiries can be directed to the corresponding author.
